# Proteomics Analysis Reveals Altered Nutrients in the Whey Proteins of Dairy Cow Milk with Different Thermal Treatments

**DOI:** 10.3390/molecules26154628

**Published:** 2021-07-30

**Authors:** Yangdong Zhang, Li Min, Sheng Zhang, Nan Zheng, Dagang Li, Zhihua Sun, Jiaqi Wang

**Affiliations:** 1 State Key Laboratory of Animal Nutrition, Institute of Animal Science, Chinese Academy of Agricultural Sciences, Beijing 100193, China; zhangyangdong@caas.cn (Y.Z.); zhengnan_1980@126.com (N.Z.); 2 Institute of Animal Science, Guangdong Academy of Agricultural Sciences, State Key Laboratory of Livestock and Poultry Breeding, Key Laboratory of Animal Nutrition and Feed Science in South China, Ministry of Agriculture and Rural Affairs, Guangdong Public Laboratory of Animal Breeding and Nutrition, Guangzhou 510640, China; minli@gdaas.cn (L.M.); lidagang@gdaas.cn (D.L.); 3 Proteomics and Metabolomics Facility, Institute of Biotechnology, Cornell University, Ithaca, NY 14853, USA; sz14@cornell.edu; 4 National Animal Husbandry Service, Beijing 100125, China; goodluck_szh@163.com

**Keywords:** whey proteins, cow milk, thermal treatments, heat sensitivity

## Abstract

Thermal treatments of milk induce changes in the properties of milk whey proteins. The aim of this study was to investigate the specific changes related to nutrients in the whey proteins of dairy cow milk after pasteurization at 85 °C for 15 s or ultra-high temperature (UHT) at 135 °C for 15 s. A total of 223 whey proteins were confidently identified and quantified by TMT-based global discovery proteomics in this study. We found that UHT thermal treatment resulted in an increased abundance of 17 proteins, which appeared to show heat insensitivity. In contrast, 15 heat-sensitive proteins were decreased in abundance after UHT thermal treatment. Some of the heat-sensitive proteins were connected with the biological immune functionality, suggesting that UHT thermal treatment results in a partial loss of immune function in the whey proteins of dairy cow milk. The information reported here will considerably expand our knowledge about the degree of heat sensitivity in the whey proteins of dairy cow milk in response to different thermal treatments and offer a knowledge-based reference to aid in choosing dairy products. It is worth noting that the whey proteins (lactoperoxidase and lactoperoxidase) in milk that were significantly decreased by high heat treatment in a previous study (142 °C) showed no significant difference in the present study (135 °C). These results may imply that an appropriately reduced heating intensity of UHT retains the immunoactive proteins to the maximum extent possible.

## 1. Introduction

Milk and dairy products are valuable foods that constitute an important part of the human diet and are consumed during the whole lifespan since they are a rich source of nutrients [1]. However, owing to its high nutritional value, milk also serves as an excellent growth medium for a variety of microorganisms [2], which may threaten the consumer’s health with food-borne illnesses. For the sake of food safety, typically, thermal treatments are commercially used. Pasteurization and indirect ultra-high temperature (UHT) treatment (≥135 °C) are the principal thermal treatments applied to milk in the dairy industry. During thermal treatments, a number of changes in nutrients have been widely studied, such as changes in proteins [3], lactose [4], and vitamins [5]. Notably, the effects of thermal treatments on the stability of milk proteins mainly focus on the denaturation of whey proteins [6].

Whey proteins have received widespread attention based on their wide range of biological activities, including antibacterial activity, fighting inflammatory disease, improving immunity, lowering blood pressure and cholesterol, promoting bone repair, etc. [7]. Thermal treatments of milk induce changes in the properties of milk whey proteins, thereby influence their function [8]. Previous studies have demonstrated that thermal treatments of whey proteins prior to enzymatic hydrolysis affect the properties of the peptides and alter their downstream bio-functionalities (e.g., angiotensin-converting enzyme (ACE) inhibitory activity and ferrous chelating capabilities) [9,10]. Furthermore, several immuneactive proteins in milk are reduced after thermal treatments [11]. Generally, whey proteins will easily undergo conformational changes with increasing temperatures, especially during UHT treatment [12]. Hence, we evaluate UHT treatment at 135 °C in the present study, in order to clarify the nutritional retention adopted by an appropriately reduced heating intensity of milk. The differences between UHT treated milk at 135 °C and a higher temperature was analyzed via the comparison of our study and a previous study (142 °C) [13].

Despite changes in the milk proteins profiles upon various thermal treatments have been extensively studied already, limited is known about the nutrient difference in low-abundance proteins from milk whey proteins with different thermal treatments and the nutritional retention by an appropriately reduced heating intensity of UHT from the perspective of proteomic analysis. Yang et al. [14] reported that 211 whey proteins (including some low-abundance proteins) were identified in five species milk using conventional shotgun proteomic techniques. Based on the analysis of differentially accumulated proteins, specific functional proteins serving as potential biomarkers in whey proteins were selected as characterization traits for a given species. Subsequently, a total of 129 bovine milk proteins quantified by proteomic study revealed the nutrient difference in milk proteins after processing via pasteurization, spray drying, and freezing [15]. In the present study, we aimed to explore the nutrient difference in the whey proteins of dairy cow milk between pasteurization (at 85 °C for 15 s) and UHT treatments (at 135 °C for 15 s), especially focusing on some low-abundance proteins using advanced proteomic techniques, in order to characterize and compare the impacts on nutrients after different thermal treatments.

## 2. Results and Discussion

### 2.1. Functional Category Analysis of Identified Proteins

**Figure 1:**
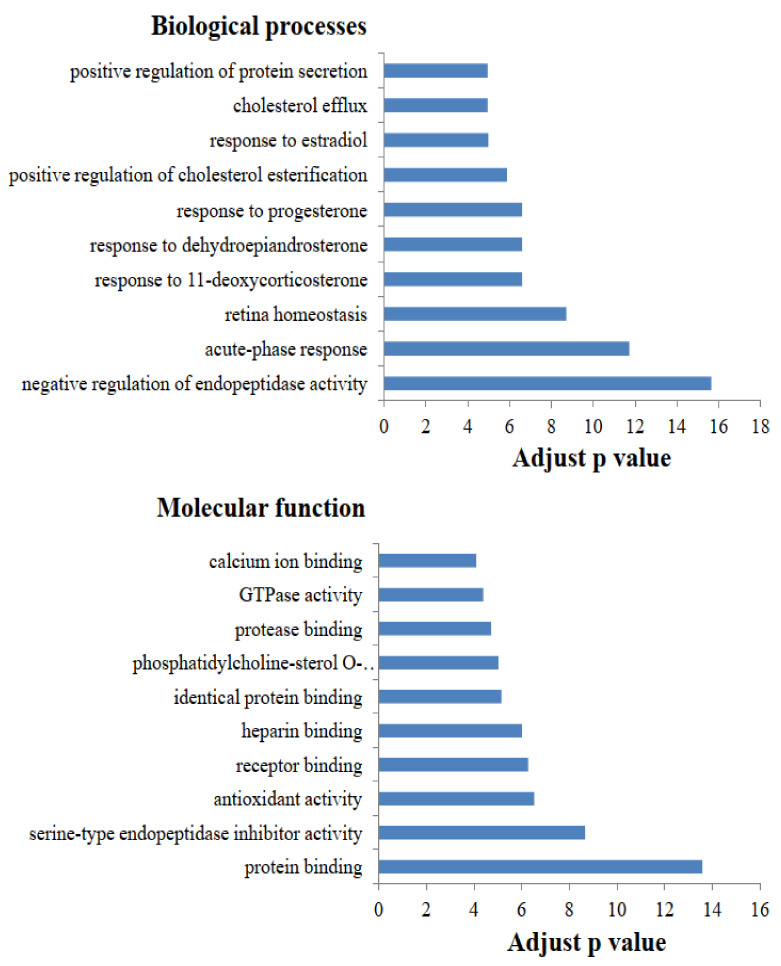
The top 10 biological processes and molecular function in the whey proteins of dairy cow milk (adjusted *p* value = −log(FDR/100)).

In the present study, 223 proteins were identified in the whey proteins using proteomics techniques (Supplemental Table S1). All identified whey proteins were further analyzed the biological processes and molecular function using gene ontology annotations. The top 10 biological processes and molecular function of whey proteins are presented in Figure 1. The identified whey proteins are involved predominantly in the biological process of negative regulation of endopeptidase activity, and the molecular function of protein binding. Sun et al. [16] reported that the largest portion of biological process was negative regulation of endopeptidase activity in cow milk, whereas that in goat milk was positive regulation of ERK1 and ERK2 cascade. Similar results were reported in a previous study that showed that the most frequent molecular function in cow and human milk was protein binding [17], which is different from the primary molecular function in goat milk [16]. The functional category analysis of whey proteins in milk provides information on the nutrient differences among animal species, thereby distinguishing specific mammalian milks [14].

### 2.2. Heat Sensitivity in Whey Proteins after Different Thermal Treatments

The results of quantification of the whey proteins are listed in Supplemental Table S2. Of these proteins, 17 were increased in abundance and 15 were decreased after UHT thermal treatment compared with pasteurization. These 32 differentially abundant proteins are presented in Supplemental Table S3. UHT thermal treatment resulted in an increased abundance of the proteins primarily involved in antigen processing and presentation (Table 1). Yang et al. [18] demonstrated that whey proteins from both Holstein and human milk are associated with antigen processing and presentation via KEGG pathway analysis. It is well known that antigen processing and presentation play essential roles in adaptive immunity responses [19]. Notably, a few relevant studies have focused on the pathway of proteoglycans in cancer; proteoglycans seem to be important macromolecules that play a critical part in the biology of various kinds of cancer [20] and participate in the innate immune system against infections [21]. This information reminds us that heat treatments might affect the nutrient content with regard to immune function in the whey proteins.

Thermal treatment of milk is an essential process of milk production adopted by the dairy industry. In this process, the functionality and molecular structure of milk proteins undergo irreversible changes [22]. Thus, the nutrient difference due to the changes in whey proteins with heat treatment might be associated with differences in the amino acid sequence, spatial structure, and post-translational modifications of the proteins involved [15]. Fifteen heat-sensitive proteins identified after UHT thermal treatment are listed in Table 2. Further research is necessary to provide insights into the mechanism of heat sensitivity for these important and heat-sensitive proteins. Some of these proteins are connected with biological immune functionality, such as Ig heavy chain [23], monocyte differentiation antigen CD14 [13], and C4b-binding protein [24]. With the support of a previous study [13], we further demonstrated that UHT thermal treatment results in a partial loss of immune function in the whey proteins of dairy cow milk. Interestingly, compared with this previous study [13], the proteins significantly decreased by high heat treatment (such as lactoperoxidase and lactoperoxidase) showed no significant difference in the current study (Supplemental Table S2). The results may imply that an appropriately reduced heating intensity of UHT (135 °C vs. 142 °C) retains immunoactive proteins to the maximum extent possible.

## 3. Materials and Methods

### 3.1. Milk Sampling and Pre-Treatment

Milk samples, pasteurized (*n* = 3) and UHT (*n* = 3), were obtained from a commercial dairy plant. The raw milk was the same for the pasteurization process and UHT treatment in each trial, and three replicated trials were undertaken using different batches of raw cow milk. Raw milk was cooled to under 4 °C and stored in a raw milk reception silo, then subjected to commercial pasteurization (85 °C for 15 s, Shanghai Nanhua Transducer Manufacture Co., Ltd., Shanghai, China) and UHT (135 °C for 15 s, FLEX-NG, Tetra Pak. Pully, Sweden) within 24 h of milking. The weight of the first batch of raw milk was 40.6 t, which was collected from more than 1500 dairy cows, then 9.6 t of milk was used for the pasteurization process and 31 t milk was used for UHT treatment. The weight of the second batch of raw milk was 48.8 t, which was collected from more than 1500 dairy cows; 8.3 t of milk was used for the pasteurization process and 40.5 t of milk was used for UHT treatment. The weight of the third batch of raw milk was 34.6 t, which was collected from more than 1200 dairy cows; 8.9 t of milk was used for pasteurization process, and 25.7 t of milk was used for UHT treatment.

Milk samples (4 mL) were centrifuged at 4 °C and 4000× *g* for 20 min, and the upper fat was manually removed to obtain skimmed milk; the procedure was repeated twice. The pH of each skimmed sample (3 mL) was adjusted to 4.6 by acetic acid under pH meter control, then 90 μL of 3.3 M sodium acetate was supplemented and centrifuged for 30 min at 14,000× *g*; the supernatant was obtained as pH 4.6 soluble protein samples. A 500 μL aliquot of the supernatant was precipitated with 2.5 mL precooled (−20 °C) phosphate-containing 20 mM DTT saturated acetone overnight at −20 °C. After centrifugation to remove the supernatant at 4 °C and 15,000× *g* for 10 min, the precipitate was washed with precooled (−20 °C) 80% acetone, then evaporated naturally in a ventilated kitchen.

### 3.2. Proteomics Treatment and Analysis

Protein pellets were re-suspended and denatured in 0.1 M phosphate buffer, pH 7.0, 5.2 M urea, 1.7 M thiourea, 0.27% SDS, 2% chaps (*w*/*v*), and 0.2% TritonX-100 (*v*/*v*). Further experimental operations were conducted on the basis of Thermo Scientific’s TMT10plex Mass Tag Labeling Kits with a slight modification. A total of 70 μg of protein in each sample was reduced, alkylated, quenched, and digested according to a previous study [25]. An aliquot of 20 μg of protein in each sample was adjusted to pH 3 using formic acid, while the remaining 50 μg protein aliquot was used for digestion and TMT labeling. The samples were first reduced with 20 mM TECP for 1 h at room temperature, alkylated with 20 mM iodoacetamide for 1 h in the dark, and then quenched by the addition of 20 mM Dithiothreitol (DTT). The alkylated proteins were precipitated by adding 6 volumes of ice-cold acetone and incubating at −20 °C overnight, then reconstituted in 50 µL of 100 mM triethylammoniumbicarbonate. Each sample was digested with 2.5 μg of trypsin for 18 h at 35 °C. The TMT 10-plex labels were conducted as follows. The tryptic peptides from six samples of milk from the different thermal treatments (pasteurized 1, pasteurized 2, pasteurized 3, UHT 1, UHT 2, and UHT 3) were mixed with each tag, named 128C-tag, 128N-tag, 129C-tag, 129N-tag, 130C-tag, and 130N-tag, respectively. After checking label incorporation using Orbitrap Fusion (Thermo-Fisher Scientific, San Jose, CA, USA) by mixing 1 µL aliquots from each sample and desalting with SCX ziptip (Millipore, Billerica, MA, USA), the six labeled samples were pooled together. The pooled TMT labeled sample was treated with 800 μL of 10 mM KH_2_PO_4_/5% ACN pH 3.0 and subjected to Mixed model, reversed-phase/strong cation exchange chromatography using Oasis@ MCX 1CC (30 mg) extraction cartridges. The eluted tryptic peptides were evaporated to dryness and prepared for high pH reverse-phase (hpRP) chromatography with a Dionex UltiMate 3000 HPLC system [25]. The LC was conducted using a gradient of 10–45% buffer B with a flow rate of 200 μL/min. Forty-eight fractions were obtained at one-minute intervals and then pooled into six fractions at 214 nm via a multiple fraction concatenation strategy [26].

All of the fractions were prepared for nanoLC-MS/MS analysis by an Orbitrap Fusion mass spectrometer (Thermo-Fisher Scientific, San Jose, CA, USA) equipped with a nanospray Flex Ion Source using high energy collision dissociation (HCD), similar to a previous report [27]. The Orbitrap was coupled with an UltiMate 3000 RSLCnano. The operating steps of nanoLC-MS/MS were executed as previously described [25,28]. All data were searched under Xcalibur 2.2 operation software.

### 3.3. Data Processing, Protein Identification, and Bioinformatic Analysis

All raw spectra from each set of TMT 10-plex experiments were analyzed using Sequest HT software within Proteome Discoverer 1.4. The bovine sequence database from NCBI was used for database searches. The default search setting used for protein identification and quantification was as described in Feng et al. [29]. Each protein identified with at least two unique peptides can be considered a confident identification. The identified proteins were analyzed by one-way ANOVA using SPSS software. When identified proteins matched with criteria *p* < 0.05 plus fold change >1.2, they were considered differentially expressed proteins [30]. The false discovery rate (FDR) of the *p* value was controlled by the Benjamini–Hochberg procedure. Functional category analysis of identified proteins was performed using the gene ontology (GO) annotation software (http://david.abcc.ncifcrf.gov/home.jsp, accessed on 11 March 2021). Subsequently, the Kyoto Encyclopedia of Genes and Genomes (KEGG; https://www.genome.jp/kegg/ accessed on 11 March 2021) was used to analyze and classify proteomics data.

## 4. Conclusions

Thermal treatments of whey proteins prior to enzymatic hydrolysis affect the properties of the peptides and alter their downstream bio-functionalities. In the current study, 223 whey proteins from dairy cow milk after different thermal treatments were confidently identified and quantified using advanced in-depth proteomics techniques. The results showed the nutrient differences in the whey proteins of dairy cow milk between pasteurized and UHT milk, thereby yielding data on the appropriate thermal treatments to use for raw milk. Particularly, we found that UHT thermal treatment resulted in a decreased abundance of 15 heat-sensitive proteins. Some of these proteins are related to biological immune functionality, suggesting that UHT thermal treatment results in a partial loss of immune function in the whey proteins of dairy cow milk. Moreover, further research is underway to shed new light on the mechanism of heat sensitivity in these proteins, and the advantage of the appropriately reduced heating intensity of various milk products.

## Figures and Tables

**Table 1:** Up-regulated and down-regulated proteins identified by KEGG enrichment analysis after UHT thermal treatment.

KEGG Pathway Name	Protein Name	UHT/Pasteurization Fold Change
Antigen processing and presentation	Protein disulfide-isomerase A3 precursor	1.29
	Cathepsin L1 precursor	1.31
	Beta-2-microglobulin	1.23
Lysosome	Cathepsin L1 precursor	1.31
	Prosaposin precursor	0.82
	Cathepsin D precursor	0.81
Phagosome	Cathepsin L1 precursor	1.31
	Ras-related C3 botulinum toxin substrate 1 precursor	1.21
	Monocyte differentiation antigen CD14 precursor	0.78
Proteoglycans in cancer	Cathepsin L1 precursor	1.31
	Ras-related C3 botulinum toxin substrate 1 precursor	1.21
	Metalloproteinase inhibitor 3 precursor	0.83

**Table 2:** Fifteen heat-sensitive proteins identified after UHT thermal treatment.

Protein Name	UHT/Pasteurization Fold Change
Alpha-S2-casein precursor	0.70
Renin receptor precursor	0.74
Solute carrier family 28 member 3 isoform X1	0.74
Ig heavy chain Mem5-like, partial	0.78
Monocyte differentiation antigen CD14 precursor	0.78
Ras-related protein Rab-18	0.79
Protein kinase C-binding protein NELL2 precursor	0.81
Cathepsin D precursor	0.81
Prosaposin precursor	0.82
Ras-related protein Rab-11A	0.82
C4b-binding protein alpha chain isoform X6	0.82
Alpha-S1-casein isoform X5	0.82
Metalloproteinase inhibitor 3 precursor	0.83
Cysteine-rich secretory protein 3 precursor	0.83
Alpha-enolase isoform X1	0.83

## Data Availability

Not applicable.

## Supplementary Materials

Supplementary materials are available online, Table S1: The 223 whey proteins identified in dairy cow milk, Table S2: Quantification information on the whey proteins in dairy cow milk, Table S3: The differentially abundant proteins between pasteurized and UHT milk.

## Sample Availability

Samples of the compounds are available from Yangdong Zhang.
